# Genetic Diversity and Spatial Segregation of *Francisella tularensis* Subspecies *holarctica* in Germany

**DOI:** 10.3389/fcimb.2019.00376

**Published:** 2019-11-06

**Authors:** Sandra Appelt, Kristin Köppen, Aleksandar Radonić, Oliver Drechsel, Daniela Jacob, Roland Grunow, Klaus Heuner

**Affiliations:** ^1^Centre for Biological Threats and Special Pathogens (ZBS2), Robert Koch Institute, Berlin, Germany; ^2^Working Group Cellular Interactions of Bacterial Pathogens, ZBS2, Robert Koch Institute, Berlin, Germany; ^3^Methodology and Research Infrastructure Genome Sequencing (MF2), Robert Koch Institute, Berlin, Germany; ^4^Bioinformatics (MF1), Robert Koch Institute, Berlin, Germany

**Keywords:** *Francisella*, tularemia, canonical SNPs, genome sequencing, genetic diversity

## Abstract

*Francisella tularensis* is an intracellular pleomorphic bacterium and the causative agent of tularemia, a zoonotic disease with a wide host range. Among the *F. tularensis* subspecies, especially *F. tularensis* subsp. *holarctica* is of clinical relevance for European countries. The study presented herein focuses namely on genetic diversity and spatial segregation of *F. tularensis* subsp. *holarctica* in Germany, as still limited information is available. The investigation is based on the analysis of 34 *F. tularensis* subsp. *holarctica* isolates and one draft genome from an outbreak strain. The isolates were cultured from sample material being that of primarily human patients (*n* = 25) and free-living animals (*n* = 9). For six of 25 human isolates, epidemiological links between disease onset and tick bites could be established, confirming the importance of arthropod linked transmission of tularemia in Germany. The strains were assigned to three of four major *F. tularensis* subsp. *holarctica* clades: B.4, B.6, and B.12. Thereby, B.6 and B.12 clade members were predominantly found; only one human isolate was assigned to clade B.4. Also, it turned out that eight isolates which caused pneumonia in patients clustered into the B.6 clade. Altogether, eight different final subclades were assigned to clade B.6 (biovar I, erythromycin sensitive) and six to B.12 (biovar II, erythromycin resistant) in addition to one new final B.12 subclade. Moreover, for 13 human and 3 animal isolates, final subclade subdivisions were not assigned (B.12 subdivisions B.33 and B.34, and B.6 subdivision B.45) because official nomenclatures are not available yet. This gives credit to the genetic variability of *F. tularensis* subsp. *holarctica* strains in Germany. The results clearly point out that the given genetic diversity in Germany seems to be comparably high to that found in other European countries including Scandinavian regions. A spatial segregation of B.6 and B.12 strains was found and statistically confirmed, and B.12 clade members were predominantly found in eastern parts and B.6 members more in western to southern parts of Germany. The portion of B.12 clade members in northeastern parts of Germany was 78.5% and in southwestern parts 1.9%.

## Introduction

*Francisella tularensis* is a small, intracellular, non-motile, Gram-negative pleomorphic bacterium and the causative agent of tularemia, a zoonotic disease with a wide range of hosts (mammals, birds, amphibians, fishes, and invertebrates) (Ellis et al., 2002; Maurin and Gyuranecz, 2016; Schulze et al., 2016). Two *F. tularensis* subspecies are of clinical relevance: *F. tularensis* subspecies *tularensis* (Jellison Type A) and *F. tularensis* subsp. *holarctica* (Jellison Type B). *F. tularensis* subsp. *tularensis* is prevalent in North America, whereas the subspecies *holarctica* is found all over the northern hemisphere. The subtype A2 of subspecies *tularensis* is described to be more virulent than *F. tularensis* subsp. *holarctica* (Jellison, 1961; Farlow et al., 2005; Vogler et al., 2009a; Molins et al., 2014; Dwibedi et al., 2016). In Germany, *F. tularensis* subsp. *holarctica* is the only *Francisella* subspecies which is known to cause disease in both animals and humans. Recently, one additional *Francisella* species (Isolate W12-1067) has been identified in Germany, yet pathogenicity needs to be evaluated (Rydzewski et al., 2014; Faber et al., 2018). However, *F. tularensis* subsp. *holarctica* originates from North America or Asia from where the bacteria spread (Vogler et al., 2009a; Karlsson et al., 2013; Dwibedi et al., 2016; Hestvik et al., 2018). Additionally, it was proposed that within the postulated spread of the pathogen from east to west, Germany might be a “melting pot,” a region where strains are mixed, reassorted, and give rise to further variants (Jusatz, 1952, 1961; Faber et al., 2018). Also, phylogenetic studies have already revealed a spread of the pathogen from Scandinavia to the southern parts of Europe (Karlsson et al., 2013; Dwibedi et al., 2016).

The minimal number of bacteria needed to cause an infection in humans depends on the route of infection. Intradermal and inhalational tularemia can already be caused by 10–25 bacteria (Saslaw et al., 1961; Jones et al., 2005). Primary infection sources for humans are free-living lagomorphs (hares and rabbits), other mammals, animal carcasses, and insects (mosquitoes and ticks) and the environment (water, dust, aerosol, and soil) (Oyston and Griffiths, 2009; Maurin and Gyuranecz, 2016). A broad host species diversity was also reported in Germany (Schulze et al., 2016), and especially hunters bear a high risk of getting infected by skinning, preparing, or consuming meat of infected hares. The high rate of seropositive animals in Germany indicated that the frequency as well as the occurrence of the pathogen in the environment and wild animals might be underestimated (Jenzora et al., 2008; Gehringer et al., 2013; Kuehn et al., 2013; Muller et al., 2013; Otto et al., 2014). There might be also a high diversity of different *F. tularensis* subsp. *holarctica* strains in northeastern parts of Germany (Antwerpen et al., 2015; Schulze et al., 2016; Faber et al., 2018). For distinguishing between *F. tularensis* subsp. *holarctica* strains which display within its subspecies little genetic variation, canonical single-nucleotide polymorphisms (canSNPs) can be used (Svensson et al., 2009a; Vogler et al., 2009b; Karlsson et al., 2013; Dwibedi et al., 2016). Based on this analysis and an erythromycin-resistant/erythromycin-sensitive phenotype and genotype, *F. tularensis* subsp. *holarctica* can be subdivided into two biovars (biovar I and biovar II) and four major clades: B.4, B.6, B.12, and B.16 (Vogler et al., 2009b; Karlsson et al., 2013). These clades can be subdivided further into subclades. The subdivision into different clades and subclades is so far not performed consistently. For instance, B.12 subclade B.75 is designated as subclade and by others as clade. However, an up-to-date typing scheme, also used in this study, was recently published (Wittwer et al., 2018).

Spatial segregation of clades predominantly found in Europe (B.6 and B.12) has already been reported (Gyuranecz et al., 2012), pointing out that B.6 is primarily found in western parts of Europe and B.12 in central to eastern parts (Koene et al., 2019). Both clades are postulated to display differences in pathogenicity in lagomorphs (Origgi et al., 2014; Origgi and Pilo, 2016; Kreizinger et al., 2017; Hestvik et al., 2018). B.6 and B.12 clade members exhibit also a different resistance to erythromycin due to a mutation in the *rrl* gene (Kudelina and Olsufiev, 1980; Karlsson et al., 2016). As mentioned above, B.6 clade members are sensitive to erythromycin and B.12 clade members are resistant (Kudelina and Olsufiev, 1980; Karlsson et al., 2016).

The objective of the study presented herein was to enhance our understanding about the genetic diversity of B.6 and B.12 clade members in Germany with a specific focus on human isolates. Also, the geographical distribution pattern of B.6 and B.12 clade members was investigated. To this end, the genomes of 34 *F. tularensis* subsp. *holarctica* isolated from mainly human and animal hosts were sequenced and compared by computational analysis based on phylogenetic constructions and canSNP analysis. The analysis also includes a draft genome of a *F. tularensis* subsp. *holarctica* strain which has caused an outbreak in Germany recently (Jacob et al., 2019).

## Materials and Methods

### Bacterial Isolates

A total of 34 *F. tularensis* subsp. *holarctica* isolates from Germany were investigated, including 25 bacterial isolates from human specimens in addition to 9 isolates from samples collected from free-living animals (wild boar, raccoon dog, fox, and hare, Table 1). All bacterial strains were isolated or received from third parties to the German National Consultant Laboratory for Tularemia in human medicine between 2007 and 2018. For the isolation of *Francisella* from different sample materials, species and subspecies identification routine diagnostic tools were applied (Broekhuijsen et al., 2003; Versage et al., 2003; Jacob et al., 2011).

**Table 1:** Overview on investigated *Francisella tularensis* subsp. *holarctica* genomes from Germany.

***F. tularensis holarctica* ID**	**Year of isolation**	**Federal state ID Germany**	**Host organism**	**Clinical manifestation of pneumonia (+ yes, – no)**	**Biovar**	**Clade**	**Final subclade^**1**^**
Fth-41	2007	BB	Human	Unknown	I	B.4	–
Fth-39	2007	TH	Hare	Unknown	II	B.12	B.34/nd
A-63/63 (FDC407)	2008	BB	Fox	–	II	B.12	B.74
A-317 (FDC409)	2012	BB	Raccoon dog	Unknown	II	B.12	B.71
A-271-1 (FDC408)	2012	BB	Beaver	Unknown	II	B.12	B.75
A-702	2015	BB	Wild boar	Unknown	II	B.12	B.71
A-655	2015	B	Human	–	II	B.12	B.34/nd
A-660	2015	BW	Human	+	I	B.6	B.45/nd
A-571	2015	MV	Hare	Unknown	II	B.12	B.33/nd
A-663	2015	MV	Human	Unknown	II	B.12	B.33/nd
A-635	2015	NI	Human	+	I	B.6	B.7
A-797	2016	BW	Human	–	II	B.12	B.34/nd
A-820	2016	BY	Human	+	I	B.6	B.45/nd
A-821	2016	BY	Human	–	I	B.6	B.45/nd
A-810-1	2016	NI	Human	–	II	B.12	B.34/36
Fth-Must°	2016^DNA^	RP	Mice	Unknown	II	B.12	B.34/nd
A-988-1	2016	RP	Hare-L (lung)	–	I	B.6	B.45/51
A-988-2	2016	RP	Hare-M (spleen)	–	I	B.6	B.45/51
A-1050	2017	BB	Human	Unknown	II	B.12	B.New
A-936	2017	BW	Human^**^	–	I	B.6	B.49
A-981	2017	BW	Human^**^	+	I	B.6	B.45/nd
A-922	2017	BW	Human	–	I	B.6	B.45/nd
A-1007	2017	BY	Human	–	I	B.6	B.46/63
A-1005	2017	RP	Human^**^	–	I	B.6	B.45/nd
A-1022	2017	RP	Human	+	I	B.6	B.45/nd
A-1020	2017	RP	Human	+	I	B.6	B.45/51
A-1049	2017	SH	Human	Unknown	I	B.6	B.45/52
A-1341	2018	BB	Human^#^	–	II	B.12	B.71
A-1158	2018	BW	Human	+	I	B.6	B.45/50
A-1174	2018	BW	Human^**^	+	I	B.6	B.45/nd
A-1308	2018	BY	Human	–	II	B.12	B.34/36
A-1338	2018	BY	Hare	Unknown	II	B.12	B.33/nd
A-1183	2018	BY	Human^**^	–	I	B.6	B.45/53
A-1201	2018	BY	Human	Unknown	I	B.6	B.45/nd
A-1171	2018	NW	Human^**^	–	I	B.6	B.45/nd

Shown are the ID of the Francisella, the year of isolation, the federal state ID in which samples from patients and animals were collected, the host organism, and known clinical manifestation of pneumonia in humans as well as the biovar, the clade, and the final assigned subclade of the respective strain. BB, Brandenburg; B, Berlin; BW, Baden-Württemberg; BY, Bavaria; MV, Mecklenburg-Western Pomerania; NI, Lower Saxony; NW, North Rhine-Westphalia; RP, Rhineland-Palatinate; SH, Schleswig-Holstein; TH, Thuringia.

1 According to Wittwer et al. (2018), “nd” means no assignment of a final subclade available, currently no published reports.

° No bacterial isolate, genomic DNA only; for further details, please see Jacob et al. (2019).

** Clinical manifestation of the disease is possibly associated to tick bit.

# *Clinical manifestation of the disease seems to be connected to contact with wild boar*.

In addition, six isolates from different European countries were included: one *F. tularensis* subsp. *holarctica* strain from Lithuania (Fth-40), three *F. tularensis* subsp. *holarctica* strains (Fth-34, Fth-35, and Fth-38), and two recently isolated from hares in Austria and two isolated from ticks in Switzerland (A-328-25 and A-328-2). The isolate Fth-40 was obtained from the Lithuanian National Public Health Surveillance Laboratory. The isolates Fth-34, Fth-35, and Fth-38 were received from Germany's Federal Institute for Risk Assessment, and the tick isolates provided by the Spiez Laboratory, Bacteriology Branch, Switzerland.

### Antimicrobial Susceptibility Testing (AST) of Bacterial Isolates and *In silico* Analysis

To collect information about antimicrobial resistances of bacterial isolates to erythromycin, 26 of 34 isolates were tested using the disk diffusion method (*n* = 17) or the microdilution methods (*n* = 16). The disk diffusion method was performed for erythromycin using only two different agar plates: Mueller–Hinton agar plates with 5% sheep blood (Becton Dickinson GmbH, Heidelberg, Germany) and chocolate plates (Oxoid, Munich, Germany). The microdilution method was performed in compliance with the Clinical and Laboratory Standards Institute (CLSI) standards (Clinical Laboratory Standards Institute, 2011). For the interpretation of minimum inhibitory concentration (MIC) values, determined MICs are in general compared to defined clinical breakpoint standards. Yet, for *F. tularensis* subsp. *holarctica*, officially released breakpoints for erythromycin, are so far not available. Therefore, MIC values higher than 16 μg/ml were interpreted as resistant, corresponding to results obtained by phylogenetic analysis of genomes. Recommendations provided in World Health Organization (2007) guidelines on tularemia were followed.

For 7 of 34 *F. tularensis* subsp. *holarctica* isolates, the erythromycin resistance was assigned *in silico* according to Karlsson et al. (2016) exclusively. The erythromycin resistance of the *F. tularensis* subsp. *holarctica* isolate Fth-41 was not investigated since this is an isolate not clustering into the B.6 or B.12 clade. The outcome of the tests was compared to results obtained by phylogenetic analysis of genomes.

### Recovery of Genomic DNA for Genome Sequencing From Bacterial Isolates

DNA extraction was performed out of bacterial colony material using the QIAGEN DNeasy Blood and Tissue Kit (Hilden, Germany) following the manufacturer's instructions. DNA elution was performed in 100 μl of QIAGEN Elution Buffer (Hilden, Germany).

### Genome Sequencing

DNA quantification was performed with the Qubit™ 4 fluorometer (Invitrogen by Thermo Fisher Scientific) using the Qubit dsDNA HS assay kit (Life Technologies, Darmstadt, Germany). To generate the libraries, the NextEra XT DNA Sample Preparation Kit (Illumina, San Diego, CA, USA) was used; the library normalization step described in the manufacturer's instructions was thereby skipped. For the estimation of the DNA fragment sizes of the libraries, the Agilent 2100 Bioanalyzer was used (Agilent Technologies, Waldbronn, Germany) utilizing the High-Sensitivity DNA Analysis Kit (Agilent Technologies, Waldbronn, Germany) and electrophoresis DNA chips.

Library pool sequencing was performed in paired-end mode on a MiSeq instrument (Illumina, San Diego, CA, USA) as previously described (Jacob et al., 2019). All genome sequences have been uploaded to the European Nucleotide Archive (ENA: www.ebi.ac.uk/ena). The BioProject ID is PRJEB33006; IDs of single data sets are provided in Supplementary Table S1.

### Computational Analysis and Phylogenetic Classification

For quality trimming and adapter clipping of Illumina raw data, an in-house pipeline QCumber was used. The pipeline comprises the following tools: FastQC version 0.11 (Andrews, 2014), trimmomatic version 0.36 (Bolger et al., 2014), and KRAKEN (Wood and Salzberg, 2014). The mapping was performed with Bowtie version 2.3 using default setting parameters (Langmead, 2010). *F. tularensis* subsp. *holarctica* LVS [National Center for Biotechnology Information (NCBI) reference: NC_007880.1] was used as the reference genome for the assembly of draft genomes (Barabote et al., 2009). BAM files were uploaded into Geneious version R9.3 (Kearse et al., 2012) for further analysis. The consensus sequences of genomes were extracted and aligned using a progressive Mauve alignment for collinear genomes applying the Muscle (version 3.6; Edgar, 2004) alignment algorithm. The alignment was used for determining canSNPs (Svensson et al., 2009b; Vogler et al., 2009a; Karlsson et al., 2013) and for phylogenetic constructions. The phylogenetic tree was constructed based on entire genome sequences and in addition for comparison also on sequences of *Francisella* pathogenicity islands (FPIs) only. To generate the phylogenetic tree, the neighbor joining method for clustering was used, applying a bootstrap of 100 (Saitou and Nei, 1987). Reference genomes included in the phylogenetic reconstructions were *F. tularensis* subsp. *holarctica* OSU18 (NCBI reference: NC_017463.1) (Petrosino et al., 2006; Puiu and Salzberg, 2008), *F. tularensis* subsp. *holarctica* FSC162 (NCBI reference: PRJNA89145) (Karlsson et al., 2013), *F. tularensis* subsp. *holarctica* FSC200 (NCBI reference: NC_019551.1) (Svensson et al., 2012), *F. tularensis* subsp. *holarctica* LVS (NCBI reference: NC_007880.1) (Larsson et al., 2005), and FTNF002-00 (NCBI reference: NC_009749.1) (Haristoy et al., 2003; Barabote et al., 2009).

In addition, seven genomes of *F. tularensis* subsp. *holarctica* strains from different European countries (A-328-25, A-328-2, Fth-40, Fth-34; Fth-35, and Fth-38) were included. Genome sequences generated during the study have been uploaded to ENA (www.ebi.ac.uk/ena). The BioProject ID is PRJEB33006; IDs of single data sets are provided in Supplementary Table S1.

The Pearson chi-squared test with Yates's correction was applied to test if the geographical distribution of *Francisella* clades (clade B.12 vs. B.6) within northern and southern parts of Germany is possible (Pearson, 1900). The Yates (1934) correction was applied to prevent overestimation of statistical significance in the small dataset. To run the statistical computing, the free software R version 3.5.1 was used (Dessau and Pipper, 2008). To perform the assessment, Germany was geographically divided into a northeastern part (group 1) and a southwestern part (group 2). Group 1 comprised a total of 13 *F. tularensis* subsp. *holarctica*; of these, 11 were classified into clade B.12 and 2 were classified into clade B.6. Group 2 comprised a total of 21 *F. tularensis* subsp. *holarctica*; of these, 4 were classified into clade B.12 and 17 were classified into clade B.6.

## Results

### Genetic Diversity in Germany and Analysis of canSNP Analysis

**Figure 1:**
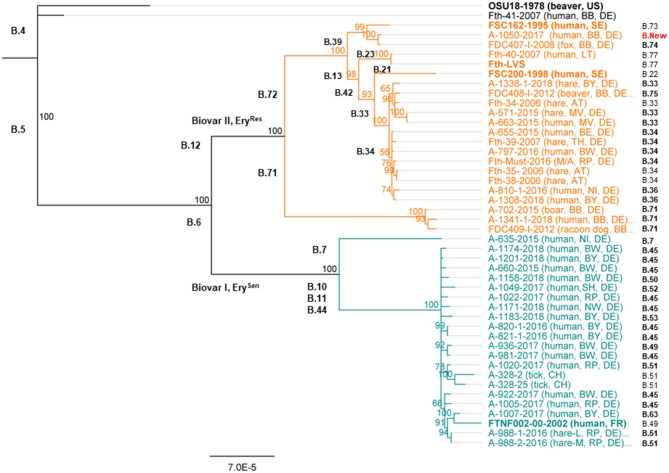
Phylogenetic relationship of *Francisella tularensis* subsp. *holarctica* in Germany. The phylogenetic analysis was based on a Mauve alignment for collinear genomes, and for the clustering, the neighbor joining bootstrap method (Fth OSU18 as an out-group) was chosen. Outlined for each genome are the identifier of the investigated *Francisella* and the year of sampling; the host organism (human or animal) and the sampling spot are indicated by the identifier of Germany's federal states. Also, the different *Francisella* clades are given in addition to the lowest assignable subclade (final subclade) for each genome. Also, reference genomes were included in the analysis; these genomes are highlighted in bold. These *Francisella* isolates come from different countries including the United States (US), France (FR), Lithuania (LT), Austria (AT), Switzerland (CH), and Sweden (SE). Germany's federal states, BB, Brandenburg; B, Berlin; BW, Baden-Württemberg; BY, Bavaria; MV, Mecklenburg-Western Pomerania; NI, Lower Saxony; NW, North Rhine-Westphalia; RP, Rhineland-Palatinate; SH, Schleswig-Holstein; TH, Thuringia.

The typing results could show that one out of 35 *F. tularensis* subsp. *holarctica* (Fth-41, Table 1) genomes clustered into clade B.4, next to the reference strain OSU18 (Figure 1). No other genome clustered into clade B.4. Altogether, 19 genomes (17 from humans and 2 from animals) were assigned to clade B.6 and 15 genomes to clade B.12 (seven from humans and eight from animals). Surprisingly, it turned out that all *F. tularensis* subsp. *holarctica* genomes associated to samples taken from patients with pneumonia (*n* = 8) clustered into clade B.6. Also, for six of 17 *Francisella* human isolates belonging to clade B.6, links between the onset of tularemia in patients and tick bites could be established (Table 1). No link between onset of tularemia and tick bites could be established for any human isolate which was assigned to clade B.12.

For clade B.6, a total of eight different lowest assignable subclades (final subclades) were determined. The dominating final subclade was B.45, followed by the final subclades B. 51, B.49, and B.63. In addition, for 10 human isolates and the draft genome of the outbreak strain (Fth-Must), a final subclade subdivision of B.45 was not assigned as no official nomenclature is available yet (Table 1).

Referring to clade B.12, six final subclades were identified, predominantly *Francisella* belonging to subclade B.33 followed by subclade B.71. In addition, a new B.12 subclade of branch B.39 could be identified, namely, B.39-New (Figure 1). For three human isolates and three animal isolates, a final subclade of subdivisions B.33 and B.34 was not assigned as no official nomenclature is available. No correlation between clade or subclades and different hosts could be identified (Table 1).

To test the reproducibility of results, biological genome duplicates were included. The results could show that these duplicates, clustered identically: A-821 and A-820, as well as A-988-2 and A-988-1 (Figure 1). The samples A-988-2 and A-988-1 were sampled from the same hare (Table 1), one from a sample taken from the spleen and one lung sample. The isolates A-821 and A-820 were both obtained from specimens coming from the same patient, taken at different time points of the infection. Interestingly, a third cluster of two *F. tularensis* subsp. *holarctica* genomes was identified. One genome from a dead hare isolate (A-663) clustered next to one human isolate (A-571) (Figure 1). Both genomes were, in terms of SNPs, identical. Furthermore, the *F. tularensis* subsp. *holarctica* genome obtained from a hunter (A-1341) grouped next to a genome from an isolate obtained from a wild boar (A-702). The wild boar *Francisella* isolate was obtained in 2015, and the hunter was infected in the same region by a wild boar in 2018. One isolate [A-317 (FDC-409)] from a raccoon dog hunted in the same region [Brandenburg (BB)] was also found to cluster in B.71 (Schulze et al., 2016), together with an isolate obtained from the hunter (A-1341) (Figure 1).

Phylogenetic constructions performed herein were based on comparison of entire genomes among each other. Analysis performed on the entire genomes was also performed on selected sequences, being those of FPIs only. The analysis was performed to gather more information on minimal input of sequence needed for phylogenetic analysis allowing drawing of correct conclusions of *Francisella* biovars, clades, and subclades (Supplementary Figure S1). It turned out that to a certain point, comparable results were achieved by relying on selected sequences only. The *Francisella* grouped into the same main clades, comprising B.4, B.6, and B.12. However, the informative and discriminative value of FPIs beyond the classification into main clades and some subclades (B.71, B.72, and B.42) seems compromised, as the assignment of other subclades could not be performed properly (Supplementary Figure S1).

### Erythromycin Resistances of Clades B.6 and B.12

The biovar typical erythromycin resistance could be confirmed by laboratory tests (microdilution method) and by *in silico* analysis for all *F. tularensis* subsp. *holarctica* strains tested. The disk diffusion methods as well as the microdilution method, yet applied without predetermined comparative clinical breakpoint values, yielded consistent results. The results obtained by phylogenetic analysis were confirmed.

### Geographical Distribution of Clades B.6 and B.12 in Germany

**Figure 2:**
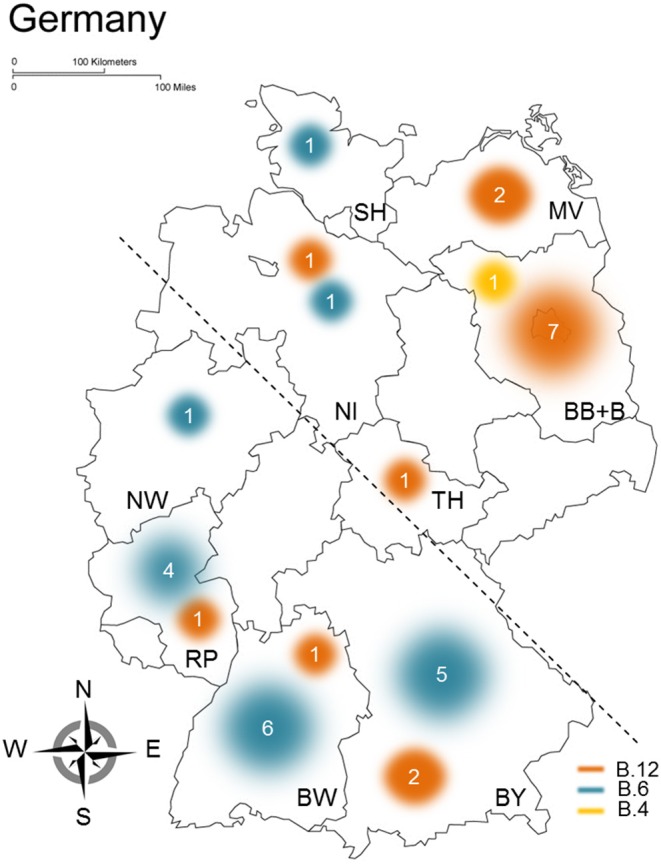
Geographical distribution of clades B.6 and B.12 in Germany. The results gathered from the analysis of different *Francisella tularensis* subsp. *holarctica* genomes are shown, outlined are assigned *Francisella* clades (yellow, B.4; orange, B.12; blue, B.6), and the sample size clustering in the respective clades is proportional to circles used for illustrating the distribution of clades in Germany. The different federal states in Germany are indicated by identifiers, BB, Brandenburg; B, Berlin; BW, Baden-Württemberg; BY, Bavaria; MV, Mecklenburg-Western Pomerania; NI, Lower Saxony; NW, North Rhine-Westphalia; RP, Rhineland-Palatinate; SH, Schleswig-Holstein; TH, Thuringia. For statistical evaluations, Germany was split into two parts (northeastern and southwestern) indicated by the dashed line.

During the investigation, differences of geographical distribution of *F. tularensis* subsp. *holarctica* clade B.6 and clade B.12 in Germany were specifically searched. A pure, perfect pattern could not be identified. However, striking the higher portion of B.12 clade members in northeastern parts of Germany, a total of 78.5% of strains of the region were assigned to clade B.12 [(group 1) 11 of 14 genomes, Figure 2], whereas in southwestern parts, the portion of B.12 clade members was 1.9% [(group 2) 4 of 21 genomes, Figure 2]. The possible geographical segregation between northeastern (group 1) and southwestern parts (group 2) of Germany is indicated by a dashed line in Figure 2. To test if the distribution of *Francisella* clades (B.12 vs. B.6 clades) within Germany is different, Pearson's chi-squared test with Yates's correction was applied. The *x*-squared was determined to 11.468 and the *p*-value to 0.000707, showing indeed that the distribution of *Francisella* clades in both groups is different. In fact, the results indicate that a geographical segregation in Germany seems to be highly likely; still further confirmation is required by testing larger sample sizes. In these lines, it could be shown that B.6 members are primarily found in southwestern parts and B.12 clade members in northeastern parts of Germany (Figure 2). *F. tularensis* subsp. *holarctica* clade B.6 was primarily found in Rhineland-Palatinate (RP), Bavaria (BY), and Baden-Württemberg (BW), whereas clade B.12 members were predominantly found in northeast Germany [Mecklenburg-Western Pomerania (MV), Brandenburg (BB), and Berlin (B)] (Figure 2). Additionally, it turned out that B.12 clade members were assigned to two additional regions [Thuringia (TH) and Lower Saxony (NI)] and B.6 members to three additional regions in Germany [North Rhine-Westphalia (NW), Schleswig-Holstein (SH), and Lower Saxony (NI)].

## Discussion

Tularemia is a rarely reported but reemerging infectious disease in Germany (Kaysser et al., 2008; Splettstoesser et al., 2009; Faber et al., 2018). A recent review has outlined aspects of the genetic diversity, epidemiological situation, and surveillance data of tularemia in Germany (Faber et al., 2018). The objective herein was to focus tighter on elucidating the genetic diversity of *F. tularensis* subsp. *holarctica* strains in Germany, with a main focus on human isolates classified into clades B.6 and B.12.

Thirty-five genomes were included in our analysis: 34 *F. tularensis* subsp. *holarctica* genomes of strains isolated mainly from humans and animal hosts and 1 draft genome from an outbreak (Fth-Must) (Jacob et al., 2019). Included in the panel are also two sets of biological duplicates (A-988-1 and A-998-2; A-820 and A-821) which clustered in the phylogenetic tree next to each other and were in terms of canSNPs identical. These findings indicate that no bias was introduced during the analysis. Also, one *F. tularensis* subsp. *holarctica* genome obtained from a strain isolated from a wild hare (A-663) clustered in the phylogenetic tree together with an isolate from a human patient (A-571). Both genomes were, in terms of SNPs, identical (Figure 1), and based on the patient report, it seems highly possible that there is an epidemiological link between both cases.

Of the 35 genomes, 34 clustered into the B.6 and B.12 clades and one genome was assigned to clade B.4. That only one of 35 analyzed genomes clustered into the B.4 clade was not surprising, as, in Europe, strains of clades B.6 and B.12 are dominant (Gyuranecz et al., 2012) and known to be present in Germany (Muller et al., 2013; Tomaso et al., 2017, 2018). The erythromycin susceptibility of biovar I (B.6 clade), as well as the erythromycin resistance of biovar II (B.12 clade), was confirmed by both experimental and *in silico* analyses. The findings are in compliance with results obtained by others showing that experimental results obtained by different means, e.g., AST using the microdilution method or disk diffusion method, can confirm *in silico* data based on phylogenetic reconstructions (Tomaso et al., 2017) or *in silico* assessment of erythromycin resistances by specifically investigating the *rrl* gene (Karlsson et al., 2016).

Besides, it turned out that all *F. tularensis* subsp. *holarctica* genomes associated to samples taken from patients with pneumonia clustered in this study into clade B.6. In hares, it was recently reported that pneumonia is significantly more common in B.6 than in B.12 cases (Koene et al., 2019). However, referring to human cases, different clinical manifestations are known to be caused by both clades (Johansson et al., 2014; Afset et al., 2015). To investigate a possible biovar-associated manifestation of pneumonia in humans would be of importance for public health matters, showing the need for analyzing genetic diversity and phylogeny of *Francisella*. In addition, the ratio of putative tick-borne tularemia in clade B.6 was surprisingly high, but this finding needs to be corroborated with more data. The ratio of almost 1:2 (8 out of 19) underlines the importance of tularemia transmitted by arthropods in Germany (Gehringer et al., 2013; Boone et al., 2015; Borde et al., 2017).

The study could clearly emphasize that a geographical segregation or clustering of *F. tularensis* subsp. *holarctica* in Germany is highly likely. Findings could show that clade B.12 members were more frequently found in northeastern parts of Germany and B.6 clade members in southwestern parts (Muller et al., 2013). A similar geographic distribution, meaning that biovar I is mainly found in Western Europe and biovar II in Northern and Eastern Europe, was already described (Kudelina, 1973; Ellis et al., 2002; Svensson et al., 2009a; Vogler et al., 2009a; Gyuranecz et al., 2012; Dwibedi et al., 2016; Karlsson et al., 2016; Origgi and Pilo, 2016; Faber et al., 2018).

Different to former analyses which were mainly based on *F. tularensis* subsp. *holarctica* isolates from brown hares, a broader host spectrum (humans and wild animals) was included next to a broader geographical scope covered (e.g., Berlin, Brandenburg) (Muller et al., 2013). But no correlation between host and subclade could be identified (Farlow et al., 2005; Pilo, 2018) as already described for *F. tularensis* subsp. *tularensis*.

Altogether, 14 different *F. tularensis* subsp. *holarctica* B.6 and B.12 final subclades were identified. For 15 isolates and the outbreak strain, the final B.12 and B.6 subclade subdivisions (subdivisions of B.33, B.34, and B.45) were not assigned because an official nomenclature is still lacking until today. Moreover, one new B.12 final subclade closely related to B.39 was identified, yet not defined. The identification of a new subclade distantly related to all other strains of subclade B.45 or B.33, and further two members of a recently identified new B.71 subcluster in Berlin-Brandenburg (Schulze et al., 2016) showed that there are still open gaps in *Francisella* phylogeny, still to be addressed by further analysis (Wittwer et al., 2018). In addition, these results show that the genetic diversity of *F. tularensis* subsp. *holarctica* strains in Germany seems to have been underestimated as initially thought (Gehringer et al., 2013; Muller et al., 2013; Schulze et al., 2016). There seems to be still room for discussions if a “sub-sub”-clades definition is needed for further phylogenetic analysis of *F. tularensis* subsp. *holarctica*. However, to find high genetic diversity gives credit to studies presuming that the diversity of tularemia in non-Scandinavian countries seems to be higher than initially expected (Chanturia et al., 2011; Gyuranecz et al., 2012; Gehringer et al., 2013; Muller et al., 2013; Antwerpen et al., 2015; Borde et al., 2017; Wittwer et al., 2018). The diversity seems even to be comparably high to Scandinavian countries known for being the source of the historical spread of the bacteria (Chanturia et al., 2011; Gyuranecz et al., 2012; Gehringer et al., 2013; Karlsson et al., 2013, 2016; Muller et al., 2013; Antwerpen et al., 2015; Schulze et al., 2016; Borde et al., 2017; Wittwer et al., 2018). For instance, a final B.6 subclade, namely, B.52, was reported to be found in Spain exclusively (Dwibedi et al., 2016) and now also assigned during the study for German *Francisella* isolates.

To conclude, the study presented herein represents a comprehensive analysis of *F. tularensis* subsp. *holarctica* strains recovered from both wild animals and human patients and is extending our current understanding about genotypic diversity of tularemia and spatial segregation in Germany.

## Data Availability Statement

The datasets generated for this study can be found in the European Nucleotide Archive, BioProject ID: PRJEB33006.

## Author Contributions

KH, RG, and DJ provided the expertise in the field of tularemia, and provided theoretical and practical advices. KH coordinated and supervised the present work. OD and AR performed whole-genome sequencing, quality trimming, and mapping. SA and KH analyzed the sequence data including the functional SNP analysis and phylogenetic constructions. RG, DJ, and SA were involved in the differential diagnostic performed on animal and human bacterial isolates as well as in the AST using the microdilution method on *Francisella* isolates. KK performed the AST using the disk diffusion method and analyzed the data. KH and SA drafted the manuscript. DJ and KK revised the manuscript critically. All authors contributed to the revision of manuscript and read and approved the submitted version.

### Conflict of Interest

The authors declare that the research was conducted in the absence of any commercial or financial relationships that could be construed as a potential conflict of interest.
