# Supplementary material for: Genetic Diversity and Spatial Segregation of Francisella tularensis Subspecies holarctica in Germany
Source: Front Cell Infect Microbiol. 2019 Nov 6;9:376. doi: 10.3389/fcimb.2019.00376 (PMC6851236; doi:10.3389/fcimb.2019.00376)
Supplement: Supplementary file 1 [file Table_1.DOCX]

***Supplementary Material***

**Title: Genetic diversity and spatial segregation of *Francisella tularensis* subsp. *holarctica* in Germany**

**Running title:** *F. tularensis* subsp. *holarctica* in Germany

Sandra Appelt^1^, Kristin Köppen^2^, Aleksandar Radonić^3^, Oliver Drechsel^4^, Daniela Jacob^1^, Roland Grunow^1^, Klaus Heuner*****^2^

^1^ Centre for Biological Threats and Special Pathogens (ZBS2), Robert Koch Institute, Berlin, Germany

^2^ Working group Cellular Interactions of Bacterial Pathogens, ZBS 2, Robert Koch Institute, Berlin, Germany

^3^ Methodology and Research Infrastructure Genome Sequencing (MF2), Robert Koch Institute, Berlin, Germany

^4^ Bioinformatics (MF1), Robert Koch Institute, Berlin, Germany

*****Correspondence:

Dr. Klaus Heuner, Cellular Interactions of Bacterial Pathogens Highly Pathogenic Microorganisms (ZBS 2), Centre for Biological Threats and Special Pathogens, Robert Koch Institute, Seestraße 10, 13353 Berlin (Germany), phone: +49 30 18754-2226

e-mail: HeunerK@rki.de

**Supplementary Figures**: 1

**Supplementary Tables**: 1

**Supplementary Figures and Tables**

**Supplementary Table S1. Listing of BioProject IDs of genome sequences generated during this study.** The sequences were uploaded on the European Nucleotide Archive (ENA: [www.ebi.ac.uk/ena](http://www.ebi.ac.uk/ena)), the ID of the BioProject IDs is PRJEB33006.

| ***F. tularensis holartica* ID** | **BioProject ID** |
| --- | --- |
| Fth-41 | in progress |
| Fth-39 | in progress |
| A-63/63 (FDC407) | in progress |
| A-317 (FDC409) | in progress |
| A-271-1 (FDC408) | in progress |
| A-702 | in progress |
| A-655 | in progress |
| A-660 | in progress |
| A-571 | in progress |
| A-663 | in progress |
| A-635 | in progress |
| A-797 | in progress |
| A-820 | in progress |
| A-821 | in progress |
| A-810-1 | in progress |
| A-988-1 | in progress |
| A-988-2 | in progress |
| A-1050 | in progress |
| A-936 | in progress |
| A-981 | in progress |
| A-922 | in progress |
| A-1007 | in progress |
| A-1005 | in progress |
| A-1022 | in progress |
| A-1020 | in progress |
| A-1049 | in progress |
| A-1341 | in progress |
| A-1158 | in progress |
| A-1174 | in progress |
| A-1308 | in progress |
| A-1338 | in progress |
| A-1183 | in progress |
| A-1201 | in progress |
| A-1171 | in progress |
| A-328-25 | in progress |
| A-328-2 | in progress |
| Fth-40 | in progress |
| Fth-34 | in progress |
| Fth-35 | in progress |
| Fth-38 | in progress |

**
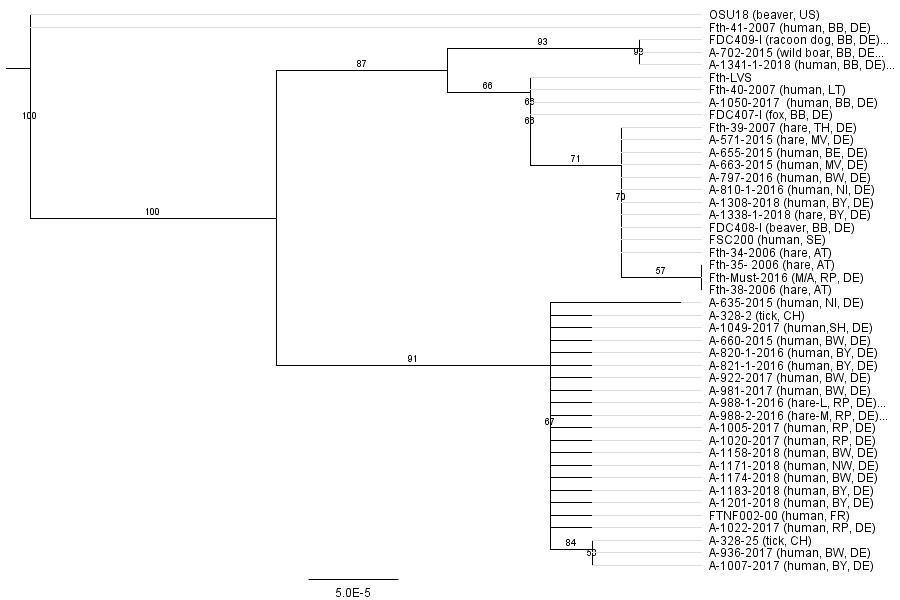
**

**Supplemantary Figure S1. Phylogenetic relationship of *F. tularensis holarctica* in Germany**. For phylogenetic construction sequences belong to Francisella Pathogenicity Islands were used for the clustering the neighbor joining bootstrap method was chosen. Outlined for each genome are: the identifier of the investigated *Francisella* and the year of sampling, the host organism (human or animal) and the sampling spot are indicated by identifier of Germany’s federal states. Marked by stars (**) are *Francisella* transmitted to host possibly by tick bites. Also reference genomes were included in the analysis; these genomes are highlighted in bold. These *Francisella* isolates come from different countries including Lithuania (LT), Austria (AT), Switzerland (CH) and Sweden (SE). Germanys federal states: BB: Brandenburg; B: Berlin; BW: Baden-Württemberg, BY: Bavaria; MV: Mecklenburg-Western Pomerania; NI: Lower Saxony; NW: North Rhine-Westphalia; RP: Rhineland-Palatinate; SH: Schleswig-Holstein; TH: Thuringia.
